# Enhancing dermal collagen density towards youthfulness: A comparative study of PCL, PLLA, and PDO thread implantation in aging rats model

**DOI:** 10.22038/ijbms.2024.80494.17428

**Published:** 2025

**Authors:** Mary Soen, Meilinah Hidayat, Wahyu Widowati

**Affiliations:** 1 Master Program in Skin Aging and Aesthetic Medicine, Faculty of Medicine, Maranatha Christian University, Bandung 40164, West Java, Indonesia, 40164; 2 Department of Nutrition, Faculty of Medicine, Maranatha Christian University, Bandung 40164, West Java, Indonesia; 3 Department of Pharmacology, Faculty of Medicine, Maranatha Christian University, Bandung 40164, West Java, Indonesia

**Keywords:** Anti-aging, COL3A1, Poly-lactic acid Polycaprolactone Polydioxanone, UVB

## Abstract

**Objective(s):**

Both intrinsic and extrinsic factors cause skin aging. Intrinsic aging is characterized by decreased collagen density, particularly collagen types I (COL1A1) and III (COL3A1), and an increase in the COL1/COL3 ratio. Extrinsic aging, primarily due to ultraviolet light exposure, leads to photoaging, which causes collagen fragmentation and reduced production, leading to skin sagging. Thread lifts, a nonsurgical method, aim to tighten the skin and stimulate collagen production using biodegradable monofilament threads such as polydioxanone (PDO), poly-lactic acid (PLLA), and polycaprolactone (PCL). This study compared the effectiveness of PDO, PLLA, and PCL threads in reversing aging by enhancing dermal collagen, reducing the COL1/COL3 ratio, and increasing COL3A1 gene expression in UVB-exposed aging model rats.

**Materials and Methods:**

Thirty female Wistar (*Rattus norvegicus*) rats were divided into six groups, and their back hair was shaved and exposed to 840 mJ/m^2^ UVB for 4 weeks. Skin biopsy specimens were assessed using Sirius Red staining to determine dermal collagen density and the COL1/COL3 ratio. Furthermore, qRT–PCR was used to examine COL3A1 gene expression.

**Results:**

PDO, PLLA, and PCL threads enhanced skin quality, similar to the young negative control group, based on parameters such as dermal collagen density, COL1/COL3 ratio, and COL3A1 gene expression. PCL thread was more active than PDO and PLLA.

**Conclusion:**

Thread implantation may result in a more youthful collagen profile than negative control and may be used to support skin anti-aging. The most effective thread was PCL compared to PDO and PLLA.

## Introduction

Aging is a natural process characterized by progressive physical and physiological changes in the body caused by cellular and tissue damage over time (1). This process manifests in various ways, including dull skin, fine lines, wrinkles, reduced muscle mass, decreased vision and hearing function, and various other health problems (2). Life expectancy among older people has notably increased in recent decades. The World Health Organization predicts that by 2050, there will be 22% more older adults worldwide. There were more people in the world over the age of 60 in 2020 than there were children under five (3). 

The two main categories of aging are intrinsic aging and extrinsic aging. Free radicals, reduced hormones, glycosylation, methylation, apoptosis, immune system weakening, and genes are some of the internal processes that contribute to aging (1, 4). Extrinsic aging is caused by environmental factors, such as pollution, sun exposure, smoking, poor diet, and stress. These factors can accelerate aging and cause faster cell damage (3, 4). 

Intrinsic and extrinsic factors cause skin aging (4). Intrinsic factors caused by increasing age decreased the density of the skin’s main collagen, namely collagen type I (COL1A1) and type III (COL3A1)(5). The decrease in type III collagen is greater than that of type I collagen; thus, the COL1/COL3 ratio increases with age (5).

The most important extrinsic factor that causes skin aging is UVB exposure, which is referred to as *photoaging *(4, 6). Exposure to UVB rays causes changes in the microscopic and macroscopic structure of the skin, resulting in thickened and dry skin and loss of elasticity (4). Collagen is particularly susceptible to damage from UVB rays because it can penetrate deeper into the skin than other types of UV rays (4, 7). UVB exposure increases matrix metalloproteinase, causing collagen degradation to increase, whereas procollagen formation decreases. 

Many facial rejuvenation techniques have been developed to restore or delay the aging process. In recent years, thread lift has become a popular minimally invasive facial rejuvenation method using monofilament threads made of biodegradable materials, which replace surgical methods (8). Thread implantation can re-stimulate collagen formation in the skin (9). This technique is considered effective for dealing with sagging skin because of its high level of safety, fast healing (1-3 days), and immediate visible effect (8, 10, 11)^.^

Various types of thread can be used for thread planting techniques, including polydioxanone (PDO), poly-lactic acid (PLLA), and polycaprolactone (PCL) threads, which are reabsorbed by the body and cause skin cell regeneration (9,12-14). PDO thread is a synthetic polymer of multiple repeating ether-ester units that is slowly hydrolyzed into a 2-hydroxyasmetic monomer and works by triggering fibroblasts to produce more collagen in the targeted area (8). PLLA is a lactic acid-derived polymer often used as orthopedic pins and sutures (12). PLLA can increase the volume of the saggy area, which helps provide a lift and restore shape to the face area (12). PCL thread is the latest monofilament suspension thread of synthetic origin (caprolactone)(15). PCL is a bioresorbable polymer often used in the medical world, such as drug-coated regulator surgical sutures. However, the PCL thread has different beneficial properties than PDO and PLLA. PDO threads can only survive in the dermis tissue and provide effects for six months, whereas PLLA lasts for 12 months, and PCL threads have the advantage of being more inert, lasting for two years, and stimulating collagen formation longer (15, 16). 

This research compared the efficacy of PDO, PLLA, and PCL threads in increasing dermal collagen, reducing the COL1/COL3 ratio, and enhancing COL3A1 gene expression in a UVB-exposed aging rat model. 

## Materials and Methods

***Animal experimental design***

**Figure 1:**
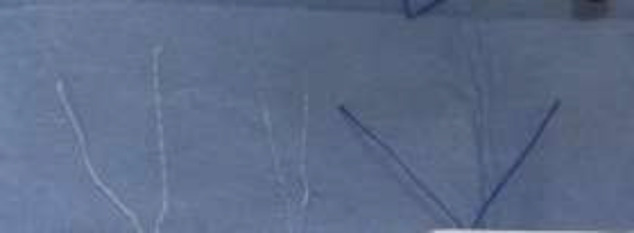
Types of threads for implantation treatments

This research was conducted using old female Wistar rats (*Ratus norvegicus* L.) aged 6-8 months for the young rat group and 16-18 months for the old rat group exposed to UVB light as an aging model. The research method was approved by the Ethics Committee of Maranatha Christian University (No. 202/KEP/XI/2023, November 27, 2023). Rats were given standard food and water *ad libitum, *weighing 300-350 grams. Rats were divided into six groups; each group consisted of 5 rats, and all dorsal hair was shaved to a size of 10 x 5 cm. The treatment group included young negative control (YNC) aged 6-8 months old rats and rats aged 16-18 months for the remaining five groups, Old Negative Control (ONC), Old Positive Control (OPC), PDO, PLLA, and PCL. The OPC and threads groups were exposed to UVB (Narrowband PL-L-36W/01/4P 1CT/25) for four weeks, with a total UVB exposure of 840 mJ/m. Thread groups were implanted with PDO (Samyang Holding Corp, Daejeon, Korea, AKL 21603121863), PLLA (Bio Co, Ltd, Incheon, Korea, AKL 21603915030), and PCL (BISTOOL, Seoul, Korea, AKL 21603120894) threads in the dermis layer, as shown in Figure 1. UVB exposure was performed three times a week: 50 mJ/cm^2 ^during the first week, 70 mJ/cm^2 ^in the second week, and 80 mJ/cm^2 ^in the third and fourth weeks (7).

***Sample collection***

After all treatments were completed, the rats were terminated by first being fasted for eight hours and then administered Ketamine (KTM-100, PT Bernofarm, Guardian Pharmatama) injection anesthesia (100 mg/kg BB) intramuscularly (17). Then, a 5x5 mm dorsal skin biopsy of the rat is performed transversely (perpendicular to the thread). Some skin samples were placed into an Eppendorf tube and stored in a deep freezer at -80 ^°^C (SimpleFreeze U500, Daihan Scientific Co, Ltd) until COL3A1 gene expression was checked. Some skin samples were stored in storage bottles and soaked in 10% formalin buffer to prepare histopathological preparations, collagen density, and COL1/COL3 ratio tests (18). 

***Histopathology assay***

Dorsal skin samples from all groups were fixed with 10% formalin and embedded in paraffin. Sections were stained with Picosirius Red to examine dermal collagen density and the COL1/COL3 ratio and were measured at 40× magnification using a bright-field microscope and polarized microscope, respectively (19).

***Dermal collagen density***

**Collagen density was obtained from histopathological preparations of rat skin with Picosirius Red staining (Pico-Sirius Red Solution, ab246832)** and were calculated by digital analysis methods using Image J software; each preparation was photographed in 15 fields of view, with an objective magnification of a bright-field microscope 40 times, and stored in JPEG form. Collagen tissue that appeared red on a light microscope (Olympus CX 22) was selected, and the result was the percentage area of collagen fraction. 

***Measurement of the COL1/COL3 ratio***

**The COL1/COL3 ratios were obtained from histopathological preparations of rat skin with Picosirius Red staining** using ImageJ software (20). Each preparation was photographed in 15 fields of view, with an objective magnification of a polarized light microscope (Olympus BX53M) 40 times, and stored in JPEG form, as shown in Figures 5 and 6. Observations were performed using a polarized light microscope to distinguish between types I and III collagen (19). When exposed to polarized light, the birefringence of type I collagen appeared reddish-orange, while that of type III collagen was bluish-green (21). **The results obtained through ImageJ are presented as the percent area of type I collagen fraction and the percent area of type III collagen fraction, after which the ratio with the percent area of type I collagen is divided by that of type III collagen (22).**

***Measurement of COL3A1 expression using qRT–PCR***

**Quantitative real-time PCR analysis of COL3A1 gene expression from rat’s skin tissue**

Total cellular RNA was extracted from skin tissue using the phenol-chloroform method. ReverTra Ace (TOYOBO-325600) was used for RT-PCR to convert the mRNAs into cDNAs (TOYOBO, BIO-65053). According to the manufacturer’s instructions, qPCR was performed using a THUNDERBIRD SYBR qPCR MIX kit (TOYOBO Bioscience, BIO-98005). Real-time PCR AriaMX 3000 (Agilent, G8830A) was used to analyze quantitative gene expression levels. At 260/280 nm, each sample’s RNA concentration and purity of each sample were assessed (Table 1). The primer sequences are presented in Table 2. The pre-incubation cycle for this system was set at 95 ^°^C for 1 min, followed by 40 cycles of denaturation at 95 ^°^C for 15 sec, followed by annealing of COL3A1 at 58 ^°^C for 45 sec with 40 cycles. Prolongated and elongated segments were performed for 30 sec at 65 ^°^C.

Quantification of COL3A1 gene expression using qRT-PCR assessed based on ΔCq values. Statistical analysis of ΔCq values obtained by reducing sample ΔCq and ΔCq housekeeping gene results*. *The control group is the OPC group, so the ΔΔ Cq value of each group is the ΔCq value of each sample minus the average ΔCq of the OPC group. The value of 2^-ΔΔ Cq^ of the OPC group was 1, and the value of 2 ^- ∆∆Cq ^of the other group was the gene expression relative to the OPC group (23).

***Statistical analysis***

All collected data were tested for normality and homogeneity using the Shapiro-Wilk and Levene’s tests, followed by the one-way ANOVA and *post hoc* LSD tests with significance based on *a P-value*≤0.05 using SPSS Software version 26.0. The Kruskal–Wallis test was performed and continued with the *post hoc* Mann-Whitney test for the not normally distributed and not homogeneous data.

## Results

***PCL threads increased dermal collagen density in aging model*** ***rats***

**Figure 2:**
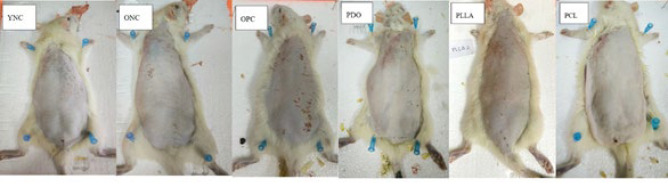
Rat skin condition after four weeks of various treatments

YNC: Young Negative Control; ONC: Old Negative Control; OPC: Old Positive Control; PCL: Polycaprolactone; PLLA: Poly-lactic acid; PDO: Polydioxanone

**Figure 3:**
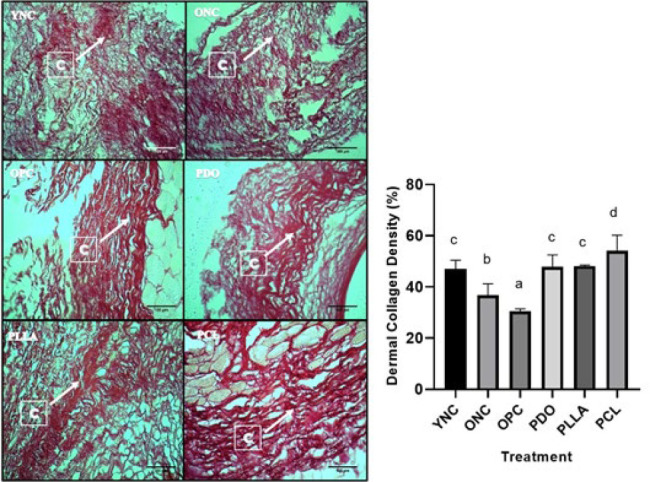
Effects of thread implantation on collagen density in UVB-exposed rats

The effect of threads on dermal collagen density in aging model rats can be seen macroscopically in Figure 2 and histologically in Figure 3. Macroscopically, the most wrinkled back skin of rats was in the OPC group (old rats and UVB exposed), while in the thread groups, the skin quality was better. In the OPC group, the skin was wrinkled, rough, and darker in color than in the other groups, and many scratch marks were found due to exposure to UVB rays causing inflammation (6, 24). The skin quality in the PCL group was the best among the thread groups, with fewer wrinkles, a smoother skin surface, and a brighter color compared with the other groups. The difference in rat age as an intrinsic aging model showed significant differences in dermal collagen density between the ONC group (old rats) and the YNC group (young rats). There was a significant decrease in average collagen density in ONC-aged rats (*P*<0.01) with YNC young rats. UVB-induced photoaging models in the OPC group also showed significant results, showing a decrease in collagen density (*P*<0.05) compared with the ONC group and very significantly with YNC. The thread implantation groups (PDO, PLLA, and PCL) showed significant increases in collagen density compared with the photoaged (OPC) and old rat (ONC) groups. Specifically, the PCL thread group exhibited significantly higher collagen density than the thread implantation and YNC groups. 

***PCL threads decreased the COL1/COL3 ratio in aging model rats***

**Figure 4:**
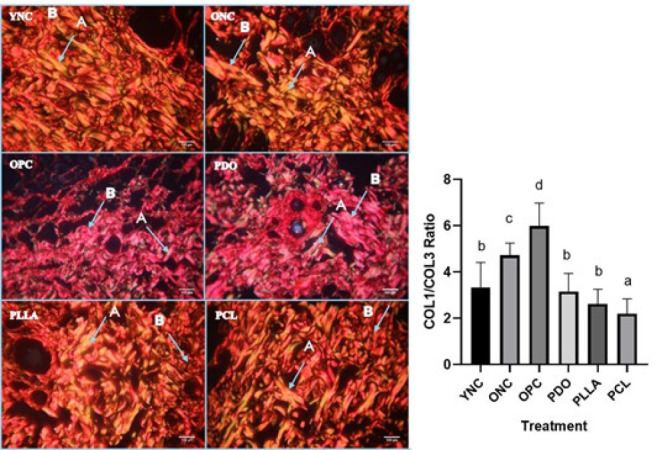
Effects of thread implantation on the COL1/COL3 ratio in UVB-exposed rats

The effect of threads on the COL1/COL3 ratio in aging model rats is shown in Figure 4. Older rats (ONC group) and UVB-induced photoaging (OPC group) exhibited significantly higher COL1/COL3 ratios than young rats (YNC group). The thread implantation groups (PDO, PLLA, and PCL) displayed significantly lower COL1/COL3 ratios than the photoaged rat group (OPC), and the PCL group had the lowest, indicating a reversal toward a more youthful collagen composition. The average COL1/COL3 ratio of the PCL group was significantly lower (*P*<0.05) than that of the YNC group. 

***PCL threads increased COL3A1 gene expression in aging model rats***

**Figure 5:**
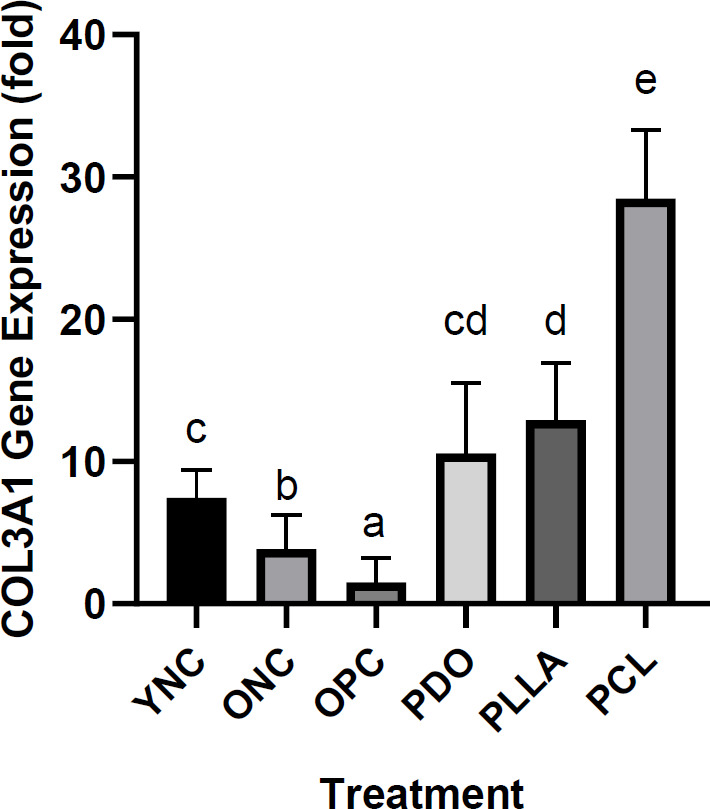
Effects of thread implantation on COL3A1 gene expression in UVB-exposed rats

The effect of thread implantation on COL3A1 gene expression in aging model rats is presented in Figure 5. The OPC group exhibited the lowest COL3A1 gene expression compared with the ONC and YNC groups. Thread implantation groups (PDO, PLLA, and PCL) showed significantly different relative COL3A1 expressions compared with the OPC and ONC groups. The PCL thread group displayed the highest relative COL3A1 expression among the thread implantation groups. 

All Coq threads (USP=2-0. PCL (left), PLLA (center), and PDO (right)

PCL: Polycaprolactone; PLLA: Poly-lactic acid; PDO: Polydioxanone

**Table 1:** RNA purity and concentrations of sample

Group	Treatment	Concentration (ng/ml)	Purity (l260/l280 nm)
YNC	Young Negative Control (6-8 months old rats, untreated)	1395	1.884
ONC	Old Negative Control (16-18 months old rats, untreated )	969,93	1.943
OPC	Old Positive Controls (old rats, UVB radiation)	1328,63	1.867
PDO	PDO (old rats, PDO thread, UVB radiation)	1190,94	1.892
PLLA	PLLA (old rats, PLLA thread, UVB radiation)	1340,45	1.842
PCL	PCL (old rats, PCL thread, UVB radiation)	1574,64	1.984

YNC: Young Negative Control; ONC: Old Negative Control; OPC: Old Positive Control; PCL: Polycaprolactone; PLLA: Poly-lactic acid; PDO: Polydioxanone

**Table 2:** Primer sequences for the analysis of COL3A1 gene expression by qRT-PCR

Gene Symbol	Forward (5^0^e3^0^)	Reverse (5^0^e3^0^)	Product Size (bp)	Annealing (°C)	Reference
COL3A1	TTTGGCACAGCAGTCCAATGTA	GACAGATCCCGAGTCGCAGA	121	58	NM_032085.1
GAPDH	GAGAAACCTGCCAAGTATG	GGAGTTGCTGTTGAAGTC	177	58	NM_001289726.1

GAPDH: Glyceraldehyde-3-Phosphate Dehydrogenase

*Left: histological image with Sirius Red staining, 40x magnification bright light microscope. C: Collagen appears as red stripes. The highest collagen density and diameter and the most intense red were observed in the PCL group. *Right: Dermal collagen density analysis. Data are presented as mean±standard deviation. Different markers (a, b, c, d) showed significant differences (*P<*0.05) between treatments of rat skin collagen density based on the post hoc LSD test (*P<*0.05)

YNC: young negative control without UVB; ONC: old negative control without UVB; OPC: old positive control exposed to UVB; PDO: PDO thread, UVB exposed; PLLA: PLLA thread, UVB exposed; PCL: PCL thread, UVB exposed

*Left: histological image with Sirius Red staining, 40x magnification polarized microscope. A: Type III collagen. B: Type I collagen. *Right: COL1/COL3 ratio analysis. Data are presented as mean±standard deviation. Different markers (a, b, c, d) showed significant differences (*P<*0.05) between treatments and the COL1/COL3 ratio of rat skin based on the post hoc LSD test (*P<*0.05)

YNC: young negative control without UVB, ONC: old negative control without UVB, OPC: old positive control exposed UVB, PDO: PDO thread, UVB exposed, PLLA: PLLA thread, UVB exposed, PCL: PCL thread, UVB exposed

*Different markers (a, b, c, cd, d, e) showed significant differences (*P<*0.05) between treatments of relative COL3A1 gene expression based on the post hoc Mann–Whitney test (*P<*0.05)

YNC: young negative control without UVB; ONC: old negative control without UVB; OPC: old positive control exposed to UVB; PDO: PDO thread, UVB exposed; PLLA: PLLA thread, UVB exposed; PCL: PCL thread, UVB exposed

## Discussion

Intrinsic and extrinsic factors cause skin aging (4). Intrinsic factors due to increasing age cause the density of the skin’s main collagen, namely collagen type I (COL1A1) and type III (COL3A1), to decrease (5). The study used female Wistar strain rats aged 16-18 months, considered equivalent to adult humans aged 45 years, to represent an intrinsic aging process (25). Young rats aged 6-8 months served as young negative controls (YNC), equivalent to humans aged 18 years. This experimental design allowed researchers to assess the effect of thread implantation on dermal collagen density in different age groups, simulating conditions comparable to human aging (25). The collagen density of the ONC-old rat group (16–18 months) was lower than that of the YNC young rat group (6-8 months), which proves a decrease in collagen density due to intrinsic aging. Intrinsic aging occurs due to several factors, such as reactive oxygen molecules, decreased growth factors, and reduced estrogen, which affect collagen breakdown (4).

This result was reinforced by the COL1/COL3 ratio results, where the COL1/COL3 ratio of the old ONC group was higher than that of the young YNC rats. With age, the production of type I collagen decreases and peaks at approximately 30 years of age. The COL1/COL3 ratio increases with age because there is a greater decrease in type III collagen than type I collagen (5, 26-28). Gao *et al*. (2023) examined the effect of increasing age on rat skin’s COL1/COL3 ratio. The COL1/COL3 ratio first increases and then stabilizes with age. The relative content of COL1 increased from week 0 to week 18, whereas the relative content of COL3 decreased with age (5).

The results of COL3A1 gene expression are also supportive, as the expression of COL3A1 in the old ONC group was lower than that in the young YNC rats. With age, COL3A1 gene expression declines (5).

The results of the three parameters showed that the age difference in the control group was meaningful, whereas age increased, collagen density decreased, the COL1/COL3 ratio increased, and COL3A1 expression decreased.

UVB exposure was used to create a photoaging model in the OPC group. The collagen density of the OPC group (exposed to UVB) is lower than that of the ONC group (not exposed to UVB). This occurs because of an imbalance between collagen production and degradation due to continuous exposure to UVB rays, where collagen degradation increases and new collagen production decreases (28, 29). When the skin is exposed to sunlight, it absorbs UV rays, producing harmful substances called Reactive Oxygen Species (ROS), which can further cause oxidative damage to cellular components, including DNA, mitochondria, and lipid membranes. ROS production triggers the activation of Mitogen-Activated Protein Kinases (MAPKs) and induction of transcription factors, such as activator protein 1 (AP-1) and Nuclear Factor-κB (NF-κB)(29). AP-1, which improves collagen breakdown, increases at least 24 hr after UV exposure. The activation of this process increases matrix metalloproteinase (MMP) expression regulation while suppressing *transforming growth factor-β *(TGF-β) signaling. As a result, collagen fragmentation occurs and collagen production decreases (28, 29). In aging skin, fibroblasts collapse due to the accumulation of degraded collagen fibers, inhibiting the formation of a healthy collagen matrix. The amount of MMP increases with age and exposure to ultraviolet radiation, resulting in increased collagen breakdown (4, 28).

The COL1/COL3 ratio parameter demonstrates the effect of UVB exposure on collagen. The results showed an increase in the COL1/COL3 ratio in the OPC group (exposed to UVB) compared with the ONC group (not exposed to UVB). The COL1/COL3 ratio in the OPC group increased because type III collagen damage was more significant than type I collagen due to UVB exposure (30). Exposure to UVB light causes changes in dermal collagen in two ways: first, stimulation of collagen breakdown, resulting in collagen breaking down into fragments and irregular breakdown. Second, procollagen biosynthesis is inhibited to reduce its content (6). 

COL3A1 gene expression was lower in the OPC group (exposed to UVB), lower than the ONC group (not exposed to UVB). Exposure to UVB light produces ROS, which causes oxidative damage to cellular components, including DNA, mitochondria, and lipid membranes, resulting in decreased expression of the COL3A1 gene and decreased type III collagen biosynthesis (28, 29).

Age differences in the intrinsic aging model (ONC group) and UVB exposure in the *photoaging *model (OPC group) showed significant differences (*P*<0.05) in collagen density, the COL1/COL3 ratio, and COL3A1 gene expression compared with the YNC group. This result proves that the treatments given to the control group in this study are meaningful. 

Thread implantation is expected to re-increase collagen density, re-decrease the COL1/COL3 ratio, and re-increase COL3A1 gene expression at a young age, as evidenced by significant differences (*P*≤0.05) in three parameters between the thread groups (PDO, PLLA, and PCL) with the OPC and ONC groups, and non-significant differences with the YNC group (young rats) in collagen density and the COL1/COL3 ratio. Expression of the COL3A1 genes in PLLA and PCL threads was significantly higher than that in the YNC group.

Implantation of biomaterials (PDO, PLLA, and PCL threads) into the body triggers a series of tissue responses involving inflammatory and wound-healing responses to injury, as well as the body’s response to foreign bodies, which are influenced by the properties of the biomaterial, composition, degradation rate, morphology, shape and size, porosity, chemistry, and surface roughness (31). Threads indirectly impact collagenesis because their presence within the dermis layer elicits a biological response in the skin, stimulating fibroblasts and further increasing the production of type I and III collagen (9, 32). In Fukaya’s (2017) study on PDO thread implantation, thread implantation can stimulate neocollagenesis through two mechanisms. The first mechanism is the occurrence of mechanical stress on skin tissue, that is, at the time of thread insertion into the dermis or subcutaneous layer. In the second pathway, threads can induce fibroblasts to undergo neocollagenesis (33).

All threads increase dermal collagen density. Still, PCL is superior to PDO and PLLA threads, where the collagen area of the PCL group is denser, the COL1/COL3 ratio is lower, and the COL3A1 expression is higher than that of PDO and PLLA. The PCL thread breakdown process is longer than that of PDO and PLLA, producing small molecular weight molecules that gradually induce collagen production by the skin (15, 34). The smaller the particles, the faster the phagocytosis, with a high degree of inflammation. PCL particle size is 25–50 μm, and its shape is round and regular, so it is protected from phagocytosis speed and minimizes inflammatory reactions. The formed collagen can be maintained on an ongoing basis (35). The advantages of PCL include rejuvenating and improving skin quality and providing long-lasting natural results. There is a low rate of side effects, and there are no reports of unexpected side effects (16, 35). Ha’s study (2022) compared different types of PDO and PCL threads in mice. The result is more collagen formation and tissue response induced by PCL threads, especially type III collagen. Meanwhile, PDO threads increased type I collagen and TGF-β. PCL threads can last longer in the tissue for more than a year, providing a more tissue-tightening effect, which is one of the most important points of face lifting (34). 

PCL thread implantation had a notable effect on altering the collagen composition toward a higher collagen density, lower COL1/COL3 ratio, and higher COL3A1 gene expression, potentially resembling a more youthful collagen profile, as observed in the YNC group. This is because the part of the skin biopsied and histological preparations were part of the skin around the thread implantation, and local inflammation activated fibroblasts and inflammatory cells. Hence, new type III collagen formation is increased in young rats compared with young YNC rats (35). The effect of thread implantation in this study was evaluated at week 4 after thread implantation; the patient had just entered the proliferation phase (31, 36). After thread insertion, the costimulatory reaction starts with subclinical inflammation caused by macrophages and multinucleated giant cells (31). Microparticle encapsulation continues, followed by collagen production by fibroblasts (31, 34, 37). Fibrillar collagen, including type III collagen, acts as the skeleton for fibroblast attachment, similar to the bone tissue scaffold method reported in the Foroutan study (2021)(38). This skeleton changes its composition over time as scar tissue strengthens. Initially, granulation tissue contains a lot of type III collagen and a little type I collagen (36, 39). During wound healing, collagen distribution and extracellular matrix remodeling are altered. The ratio of collagen I to collagen III changes with a higher amount of collagen III in the early healing phase, and collagen I gradually increases when the wound heals (36)**. **Increased type I collagen in thread implantation begins to occur during the remodeling phase, which is estimated to occur 3-4 months after implantation, and neocollagenesis and neoangiogenesis are formed to improve skin structure (35-37).

The advantage of this study is that it can be used to determine the correlation between dermal collagen density and the COL1/COL3 ratio, where thread implantation causes an increase in dermal collagen density, significantly increasing type III collagen. The control group is complete, starting from the control group of young rats, the control group of the intrinsic aging model, and the control group of the photoaging model, so that the effects of various treatments given in the study can be seen. Observations of the effect of thread implantation can be compared with a group of young rats to assess the antiaging effect of PDO, PLLA, and PCL threads in which thread implantation can increase dermal collagen, similar to that in a young animal. However, there are still several areas for improvement in this study, including the small number of samples and limited research time; if observed longer, a picture of the process of thread absorption by the body can be obtained, and other body responses that may occur. 

## Conclusion

Thread implantation may result in a more youthful collagen profile than that of the young control group and may be used to support skin anti-aging. The most effective thread type is PCL, compared with PDO and PLLA. Further research is needed to determine the long-term effects of thread implantation, from resorption to remodeling. 

## Declaration

**Authorship did not use artificial intelligence (AI) tools.**

## References

[1] Poulose N, Raju R (2014). Aging and injury: Alterations in cellular energetics and organ function. Aging Dis.

[2] Liguori I, Russo G, Curcio F, Bulli G, Aran L, Della-Morte D (2018). Oxidative stress, aging, and diseases. Clin Interv Aging.

[3] WHO Ageing and Health [Internet].

[4] Zargaran D, Zoller F, Zargaran A, Weyrich T, Mosahebi A (2022). Facial skin ageing: Key concepts and overview of processes. Int J Cosmet Sci.

[5] Gao J, Guo Z, Zhang Y, Liu Y, Xing F, Wang J (2023). Age-related changes in the ratio of Type I/III collagen and fibril diameter in mouse skin. Regen Biomater.

[6] Gromkowska-Kępka KJ, Puścion-Jakubik A, Markiewicz-Żukowska R, Socha K (2021). The impact of ultraviolet radiation on skin photoaging — review of in vitro studies. J Cosmet Dermatol.

[7] Mayangsari E, Mustika A, Nurdiana N, Samad NA (2024). Comparison of UVA vs UVB Photoaging Rat Models in Short-term Exposure. Med Arch.

[8] Bertossi D, Botti G, Gualdi A, Fundarò P, Nocini R, Pirayesh A (2019). Effectiveness, longevity, and complications of facelift by barbed suture insertion. Aesthetic Surg J.

[9] Yoon JH, Kim SS, Oh SM, Kim BC, Jung W (2019). Tissue changes over time after polydioxanone thread insertion: An animal study with pigs. J Cosmet Dermatol.

[10] Yongtrakul P, Sirithanabadeekul P, Siriphan P, Aging AF (2016). Thread lift : Classification , technique , and how to approach to the patient. Int J Med Heal Sci.

[11] Halepas S, Chen XJ, Ferneini EM (2020). Thread-lift sutures: Anatomy, technique, and review of current literature. J Oral Maxillofac Surg.

[12] Fitzgerald R, Bass LM, Goldberg DJ, Graivier MH, Lorenc ZP (2018). Physiochemical characteristics of poly-l-lactic acid (PLLA). Aesthetic Surg J.

[13] Kim JS, In CH, Park NJ, Kim BJ, Yoon HS (2020). Comparative study of rheological properties and preclinical data of porous polycaprolactone microsphere dermal fillers. J Cosmet Dermatol.

[14] Contreras C, Donado AA, Fontalvo AA (2023). Using PDO threads : A scarcely studied rejuvenation technique. J Cosmet Dermatol.

[15] Cho SW, Shim JH, Ho B, Chan S, Heo Y (2021). Efficacy study of the new polycaprolactone thread compared with other commercialized threads in a murine model. J Cosmet Dermatol.

[16] Hong G, Kim S, Park SY, Wan J, Yi K (2024). Thread lifting materials : A review of its difference in terms of technical and mechanical perspective. Clin Cosmet Investig Dermatol.

[17] Linsenmeier RA, Beckmann L, Dmitriev A V (2020). Intravenous ketamine for long term anesthesia in rats. Heliyon.

[18] Widowati W, Widyanto RM, Husin W, Ratnawati H, Laksmitawati DR, Setiawan B (2014). Green tea extract protects endothelial progenitor cells from oxidative insult through reduction of intracellular reactive oxygen species activity. Iran J Basic Med Sci.

[19] Bhutda S, Surve M V, Anil A, Kamath K, Singh N, Modi D (2017). Histochemical staining of collagen and identification of its subtypes by picosirius red dye in mouse reproductive tissues. Bio Protoc.

[20] Sharun K, Banu SA, Mamachan M, Subash A, Mathesh K, Kumar R (2023). Comparative evaluation of masson’s trichrome and picrosirius red staining for digital collagen quantification using imagej in rabbit wound healing research. J Exp Biol Agric Sci.

[21] Padilla CML De, Coenen MJ, Tovar A, Vega RE De, Evans CH, Müller SA (2021). Picrosirius red staining : Revisiting its application to the qualitative and quantitative assessment of collagen type I and type III in tendon. J Histochem Cytochem.

[22] Costa-Almeida R, Calejo I, Reis RL, Gomes ME (2018). Crosstalk between adipose stem cells and tendon cells reveals a temporal regulation of tenogenesis by matrix deposition and remodeling. J Cell Physiol.

[23] Ganger MT, Dietz GD, Ewing SJ (2017). A common base method for analysis of qPCR data and the application of simple blocking in qPCR experiments. BMC Bioinformatics.

[24] Mukherjee S, Korting HC, Roeder A (2006). Retinoids in the treatment of skin aging : An overview of clinical efficacy and safety. Clin Interv Aging.

[25] Sengupta P (2013). The laboratory rat: Relating its age with human’s. Int J Prev Med.

[26] Wang C, Rong Y hua, Guo an Z, Gang NF (2011). The content and ratio of type I and III collagen in skin differ with age and injury. African J Biotechnol.

[27] Alcaide-ruggiero L, Molina-hernández V, Granados MM, Domínguez JM (2021). Main and minor types of collagens in the articular cartilage : The role of collagens in repair tissue evaluation in chondral defects. Int J Mol Sci.

[28] Shin JW, Kwon SH, Choi JY, Na JI, Huh CH, Choi HR (2019). Molecular mechanisms of dermal aging and antiaging approaches. Int J Mol Sci.

[29] Han SH, Ballinger E, Choung SY, Kwon JY (2022). Anti-photoaging effect of hydrolysates from pacific whiting skin via MAPK/AP-1, NF-κB, TGF-β/Smad, and Nrf-2/HO-1 signaling pathway in uvb-induced human dermal fibroblasts. Mar Drugs.

[30] Varani J, Spearman D, Perone P, Fligiel SEG, Datta SC, Wang ZQ (2001). Inhibition of type I procollagen synthesis by damaged collagen in photoaged skin and by collagenase-degraded collagen in vitro. Am J Pathol.

[31] Li JJ, Zreiqat H ( 2019). Tissue response to biomaterials. Encyclopedia of Biomedical Engineering.

[32] Kim HY, Im HY, Chang HK, Jeong H Do, Park JH, Kim H Il (2023). Correlation between collagen Type I/III ratio and scar formation in patients undergoing immediate reconstruction with the round block technique after breast-conserving surgery. Biomedicines.

[33] Fukaya M (2018). Two mechanisms of rejuvenation using thread lifting. Plast Reconstr Surg Glob Open.

[34] Ha YI, Kim JH, Park ES (2022). Histological and molecular biological analysis on the reaction of absorbable thread; Polydioxanone and polycaprolactone in rat model. J Cosmet Dermatol.

[35] Christen MO, Vercesi F (2020). Polycaprolactone: How a well-known and futuristic polymer has become an innovative collagen-stimulator in esthetics. Clin Cosmet Investig Dermatol.

[36] Singh D, Rai V, K Agrawal D (2023). Regulation of collagen I and collagen III in tissue injury and regeneration. Cardiol Cardiovasc Med.

[37] Carnicer LA, Shao TC, George G M, Damiano G B (2021). Foreign body reaction to implanted biomaterials and its impact in nerve neuroprosthetics. Front Bioeng Biotechnol.

[38] Foroutan S, Hashemian M, Khosravi M, Nejad MG, Asefnejad A, Saber-Samandari S (2021). A porous sodium alginate-CaSiO3 polymer reinforced with graphene nanosheet: Fabrication and optimality analysis. Fibers Polym.

[39] Karsdal MA, Leeming DJ, Henriksen K, Bay-Jensen AC (2016). Biochemistry of Collagens, Laminins and Elastin: Structure, Function and Biomarkers. Biochemistry of Collagens, Laminins and Elastin: Structure, Function and Biomarkers.

